# Premature Mortality, Risk Factors, and Causes of Death Following Childhood-Onset Neurological Impairments: A Systematic Review

**DOI:** 10.3389/fneur.2021.627824

**Published:** 2021-04-09

**Authors:** Jonathan A. Abuga, Symon M. Kariuki, Samson M. Kinyanjui, Michael Boele van Hensbroek, Charles R. Newton

**Affiliations:** ^1^Kenya Medical Research Institute (KEMRI-Wellcome Trust Research Programme), Clinical Research (Neurosciences), Kilifi, Kenya; ^2^Global Child Health Group, Emma Children's Hospital, Academic Medical Centre, University of Amsterdam, Amsterdam, Netherlands; ^3^Department of Public Health, Pwani University, Kilifi, Kenya; ^4^Department of Psychiatry, University of Oxford, Oxford, United Kingdom; ^5^Nuffield Department of Medicine, University of Oxford, Oxford, United Kingdom

**Keywords:** neurological, neurodevelopmental, disability, impairment, mortality, children

## Abstract

**Background:** Neurological impairment (NI) and disability are associated with reduced life expectancy, but the risk and magnitude of premature mortality in children vary considerably across study settings. We conducted a systematic review to estimate the magnitude of premature mortality following childhood-onset NI worldwide and to summarize known risk factors and causes of death.

**Methods:** We searched various databases for published studies from their inception up to 31st October 2020. We included all cohort studies that assessed the overall risk of mortality in individuals with childhood-onset epilepsy, intellectual disability (ID), and deficits in hearing, vision and motor functions. Comparative measures of mortality such as the standardized mortality ratio (SMR), risk factors and causes were synthesized quantitatively under each domain of impairment. This review is registered on the PROSPERO database (registration number CRD42019119239).

**Results:** The search identified 2,159 studies, of which 24 studies were included in the final synthesis. Twenty-two (91.7%) studies originated from high-income countries (HICs). The median SMR was higher for epilepsy compared with ID (7.1 [range 3.1–22.4] vs. 2.9 [range 2.0–11.6]). In epilepsy, mortality was highest among younger age groups, comorbid neurological disorders, generalized seizures (at univariable levels), untreatable epilepsy, soon after diagnosis and among cases with structural/metabolic types, but there were no differences by sex. Most deaths (87.5%) were caused by non-epilepsy-related causes. For ID, mortality was highest in younger age groups and girls had a higher risk compared to the general population. Important risk factors for premature mortality were severe-to-profound severity, congenital disorders e.g., Down Syndrome, comorbid neurological disorders and adverse pregnancy and perinatal events. Respiratory infections and comorbid neurological disorders were the leading causes of death in ID. Mortality is infrequently examined in impairments of vision, hearing and motor functions.

**Summary:** The risk of premature mortality is elevated in individuals with childhood-onset NI, particularly in epilepsy and lower in ID, with a need for more studies for vision, hearing, and motor impairments. Survival in NI could be improved through interventions targeting modifiable risk factors and underlying causes.

## Introduction

Neurological impairments (NI) are a group of disorders resulting from damage to or dysfunction of the central nervous system (1–3). The most prevalent domains of NI include epilepsy, cognitive, sensorineural, and motor impairments (4, 5). The burden of NI varies greatly between and within regions and countries, which is attributed to epidemiological and demographic transitions (6–10). For example, improved child survival and persistence or emergence of risk factors for NI have increased the burden in older children and adolescents in LMICs (11–13).

Current evidence suggests an increased risk of premature mortality or reduced life expectancy among individuals with NI and disability (14–19). For instance, the risk of premature mortality is 2–3 times higher among people with epilepsy compared with the general population (16, 19); the risk is highest in LMICs (16) and childhood-onset seizures (19). The risk of mortality is also higher in structural/metabolic, untreated and intractable epilepsy. The causes of death in epilepsy include: (i) sudden unexplained death in epilepsy (SUDEP); (ii) accidents/burns; or (iii) acute/chronic infectious or non-infectious disease (16, 19), but it is unclear if mortality for other domains of NI is related to these or other different causes. Cohort studies are logistically intensive to conduct, which can influence the extent to which mortality is examined following NI across the world, particularly in LMICs.

We conducted a systematic review to: (i) estimate the magnitude of premature mortality in individuals with childhood-onset NI; (ii) summarize known risk factors; and (iii) describe causes of premature mortality among individuals who died. This evidence is required to inform medical and community-based interventions that might improve the survival and quality of life for individuals with NI and disability.

## Methods

We used the Preferred Reporting Items for Systematic Reviews and Meta-analyses (PRISMA) guidelines (20) and the Centre for Reviews and Dissemination (CRD) recommendations for undertaking reviews in healthcare (21) for the searches, identification, appraisal of eligible studies, synthesis, and reporting of findings in this review. A protocol is registered in the international prospective register of systematic reviews (PROSPERO-registration number CRD42019119239) (22).

### Search Strategy and Eligibility Criteria

A search was conducted in the PubMed, EMBASE, and Scopus databases for cohort studies from database inception up to 31st October 2020 using terms in three groups: (i) neurologic impairments, cognitive impairments or intellectual disability, motor impairments, visual impairments, hearing impairments and epilepsy; (ii) mortality, death or survival; (iii) risk factors, predictors or causes of death (see Table 1 for details of the search strategy in PubMed).

**Table 1:** Searches in PubMed.

**Database**	**Search terms**
PubMed	(((excess mortality OR long-term survival OR life expectancy OR premature death OR death OR premature mortality OR survival) AND (neurodevelopmental disorders [MeSH Terms] or neurologic impairment OR cognitive disability OR motor impairment OR visual impairment OR hearing impairment OR epilepsy)) AND (risk factors OR causes of death OR predictors of mortality)) AND (cohort*) **Filters:** Humans

Two reviewers reviewed the retrieved citations in a two-stage process. In the first stage, the first reviewer (JA) reviewed all titles and available abstracts to identify relevant studies. The second reviewer (SK) independently reviewed 30% of the titles and abstracts; both reviewers compared their lists and resolved disagreements by consensus. In the second stage, both reviewers assessed whether the identified articles met the inclusion criteria.

### Inclusion and Exclusion Criteria

We included: (i) original cohort studies of mortality following children with NI in five domains (epilepsy and impairments in cognitive, hearing, vision, and motor functions); (ii) studies with a childhood-onset or diagnosis of impairment (between the ages 0–19 years); (iii) studies reporting all-cause mortality as the primary outcome; and (iv) studies with an appropriate comparison group such as matched controls or the general population. We excluded studies with adulthood-onset of NI (≥20 years), studies of mental and psychiatric problems, studies without an appropriate comparison group, those reporting the same data in different papers, reviews, editorials and studies reported in other languages that could not easily be translated into English.

### Definition of Neurological Impairments and Data Extraction

An assessment was done on whether the definitions of NI in each study were in alignment with the International Classification of Diseases (ICD), the Diagnostic and Statistical Manual for Mental Disorders (DSM), or both with the versions dependent on the year of study. Childhood-onset epilepsy was defined according to the International League Against Epilepsy (ILAE) as the presence of two or more unprovoked seizures occurring within 12 months identified before the age of 18 years (23). Individuals with cognitive impairment, hereafter referred to as intellectual disability (ID), refer to those who had IQ scores <70 or z-scores < −3 based on age-appropriate neuropsychological tests and age-inappropriate adaptive skills with a childhood-onset (24). Historically, cognitive impairment has been conceptualized as structural or functional limitations based on the medical model (2, 25); however, recent definitions have used the term ID to depict the misfit between contextual demands and the person's capabilities (3, 26). Motor impairments referred to limitations in muscle control, movement, or mobility, or complete absence of motor functioning based on valid criteria such as the Gross Motor Function Classification System (GMFCS) (27). Hearing impairment was defined as hearing loss >25–30 dB in the best hearing hear (28) and vision impairment as a deficit in sight presenting with visual acuity worse than 6/12 (29). We extracted data on study setting, population characteristics, cohort sizes, duration of follow-up, comparative measures of mortality risk such as mortality rate ratios (MRR), hazard ratios (HR) and standardized mortality ratios (SMR), and risk factors and causes of death as reported in the individual studies.

### Quality of Studies

Guidelines from the Joanna Briggs Institute's (JBI) critical appraisal checklist for cohort studies were used (30) and the Strengthening the Reporting of Observational Studies in Epidemiology (STROBE) checklist (31) to appraise the methodological quality of the included studies. Our emphasis on quality was focused on: (i) reliability/sensitivity of NI diagnosis and case ascertainment; (ii) sensitivity/reliability of mortality case and cause of death ascertainment; (iii) representativeness of the study population; (iv) risk of bias e.g., selection bias; (v) follow-up duration; and (vi) use of appropriate analytic methods. We grouped each report into an aggregate score of four classes: class 1 studies represent those studies with an overall score between 75 and 100%; class 2 studies scored between 50 and 74%; class 3 studies scored between 25 and 49% and class 4 studied scored below 25% representing the weakest evidence.

### Synthesis of Evidence

We estimated the overall risk of premature mortality separately for each domain of NI because of heterogeneity in the diagnosis of each, and that each impairment has a unique underlying process and prognosis. Summary measures such as the median and range were used for descriptive analysis of SMR, MRR, and HR reported in the included studies. Similarly, we summarized the measures of effect on mortality for each risk factor and cause-specific proportionate mortality per domain of NI. Overall estimates of mortality from primary studies could not be combined in a meta-analysis because there was very high heterogeneity within and between the included studies (32).

## Results

### Search Results

**Figure 1:**
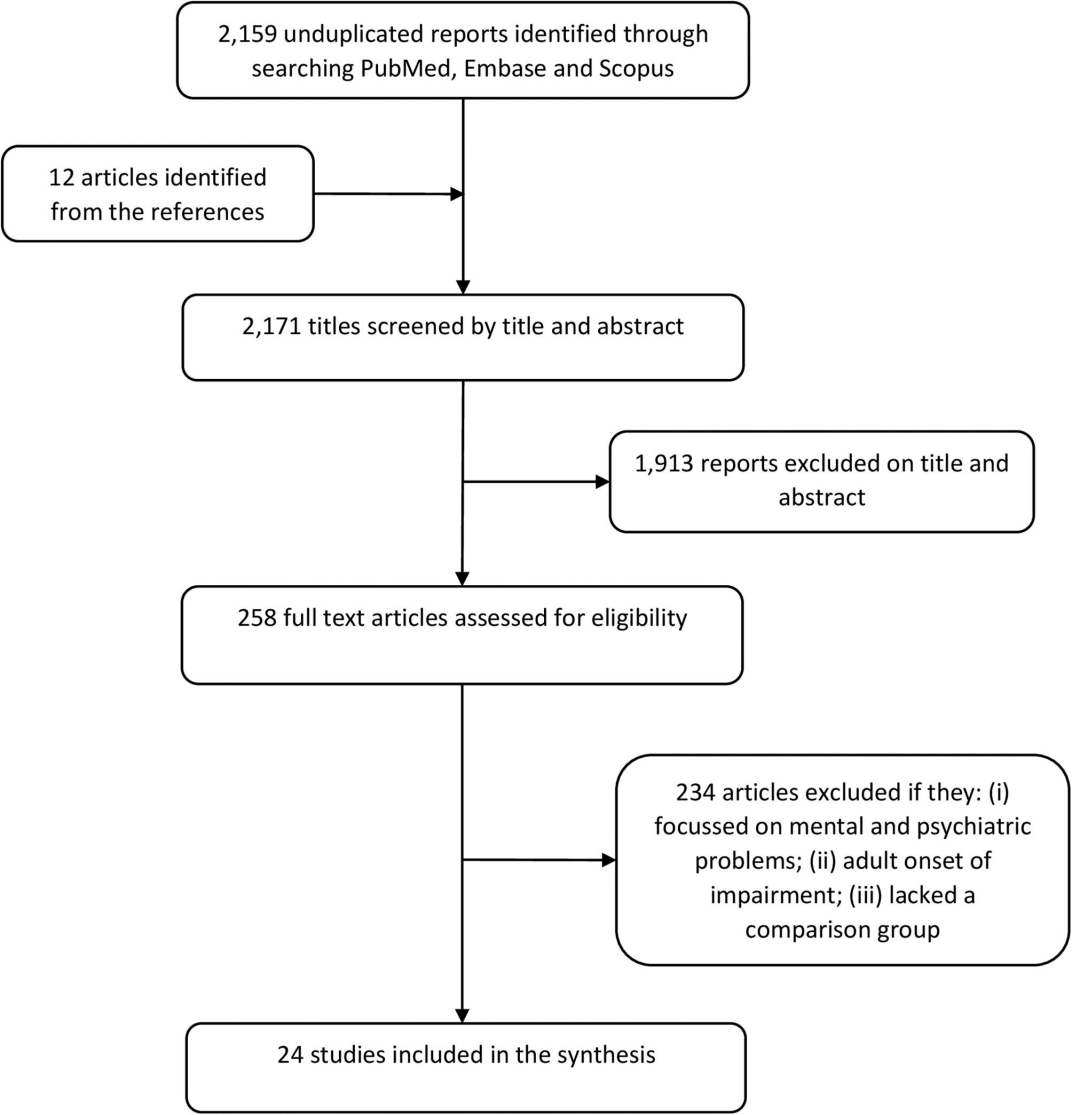
PRISMA flowchart summarizing the systematic literature search.

The results of the systematic search are in Figure 1. A total of 24 studies met the inclusion criteria of which 9 (37.5%) were on epilepsy, 10 (41.7%) on ID, 3 (12.5%) on motor-related impairments or cerebral palsy (CP), 1 (4.2%) on vision impairment and 1 on multiple domains of NI (Supplementary Table 1). Fourteen studies (58.3%) reported findings from population-based cohorts and 10 (41.7%) from clinical cohorts. Of the 24 studies, 5 (20.8%) were prospective in design while the rest (79.2%) identified study participants retrospectively. Europe provided 11 (45.8%) studies, North America 7 (31.8%) studies, Australia 4 (18.2%) studies, Asia one (4.5%) study, and Africa one study (Supplementary Table 1).

### Quality of Studies

Most studies (63%) were classified as class 1 or excellent quality (Supplementary Table 1) and the median quality score for all studies was 91% (range [55–100]). The median quality score was similar for population-based studies and clinical cohort studies (91% [55–100] vs. 87% [64–100]; *p* = 0.76). The median quality score for retrospective cohort studies was also comparable with the median score for prospective studies (91% [55–100] vs. 100% [82–100], *p* = 0.21).

### Epilepsy

#### Overall Risk of Mortality

The median SMR was 7.1 (range 3.1–22.4) for children with epilepsy (Table 2), and none of these studies originated from LMICs. The median SMR for clinical cohort studies (33–35) was 7.5 (range 7.0–22.4) and 6.8 (range 3.1–9.0) for population-based studies (36, 37, 39, 41). One study (38) reported a MRR of 14.9 (95% CI 13.9–16.1) and the other study (40) a hazard ratio of 3.8 (95% CI 3.1–4.7).

**Table 2:** General study characteristics, overall risk, and mortality by age and sex in children with epilepsy.

**Study**	**Country or State**	**Population characteristic**	**Percent quality score (Class)**	**Cohort size**	**Follow-up (years)**	**Measure of mortality (95% CI)**	**Age-specific mortality ratios (95% CI)**	**Sex-specific** **mortality ratios** **(95% CI)**	
								**Males**	**Females**
**Clinical-based cohort studies**									
Ackers et al. (33)	England and Wales	Incident cases aged 0–18 years	64 (2)	6,190	^a^13	SMR 22.4 (18.9–26.2)	SMR of 42.4 (95% CI 33.3–53.2) for 2–11 years; 13.8 (10.4–18.0) for 12–18 years; 20.9 (13.2–31.3) for <2 years	SMR 19.4 (15.5–23.9)	27.1 (20.9–34.5)
Berg et al. (34)	Connecticut, USA	Incident cases aged <16 years	82 (1)	613	^b^7.9	SMR 7.5 (4.38–12.99)	Not reported	2.0%	2.3% (*p* = 0.77, SMR not estimated)
Callenbach et al. (35)	The Netherlands	Incident cases	99 (1)	472	^a^5	SMR 7.0 (2.4–11.5)	Not reported	SMR 6.6 (2.2–15.5)	7.4 (2.0–19.0)
**Population-based cohort studies**									
Autry et al. (36)	Atlanta, USA	Incident cohort 10 years	99 (1)	688	^a^26	SMR 3.1 (2.39–3.98)	SMR of 0.3 (0.07–0.93) for <1 year; 6.0 (3.0–10.7) for 1–4 years; 10.1 (5.2–17.6) for 5–9 years; 16.7 (9.5–27.1) for 10–14 years; 3.1 (1.5–5.7) for 15–19 years; 3.3 (1.6–5.9) for 20–24 years; and 1.7 (0.0–9.7) for 25–34 years.	SMR 3.0 (2.1–4.0)	3.4 (2.1–5.1)
Camfield et al. (37)	Nova Scotia, Canada	Incident cases <17 years	99 (1)	686	^b^13·9	SMR 7.1 (3.2–10.9)	Age at onset 1–5 years vs. <1 year (RR_ADJ_ 1.5, 95% CI 0.6–3.9), and 6–16 years (RR_ADJ_ 1.7, 95% CI 0.5–6.1).	Multivariable relative risk (girl vs. boy) 1.3 (0.6–2.9)	
Christensen et al. (38)	Aarhus, Denmark	Incident cases	99 (1)	25,244	^b^13.7	MRR 14.9 (13.9–16.1)	Short-term mortality (<1 year) in epilepsy with an onset before 5 years (MRR 41.5, 95% CI 35.4–48.3); long-term mortality (>1 year) (MRR 21.6, 95% CI 19.5–23.8).	Cumulative mortality 20 years after first epilepsy diagnosis 7.6% (6.8–8.4)	Cumulative mortality 5.8% (5.1–6.5)
Nickels et al. (39)	Rochester, MN, USA	Incident cases <18 years	73 (2)	467	^b^7.9	SMR 9.0 (5.4–14.4)	11 of 16 (69.8%) deaths occurred among children aged 1 month−10 years; 4 (25%) deaths occurred in the 10–19 years age group; one (6.3%) death occurred in the 20 years or older age group.	Not reported	Not reported
Selassie et al. (40)	South Carolina, USA	Incident cases ages <19 years	91 (1)	13,098	^a^11	HR 3.8 (3.1–4.7)	Mortality in children aged 13–18 years vs. 0–5 years (HR 1.5, 95% CI 1.2–1.9); 6–12 years (HR 0.9, 95% CI 0.7–1.1).	Males vs. females HR 1.28 (1.08–1.51)	
Sillanpaa and Shinnar (41)	Turku, Finland	Incident and prevalent cases <16 years	99 (1)	245	^b^40	SMR 6.4 (5.9–7.0)	Age at onset (<2 years vs. ≥ 2 years): HR 1.7 (0.8–3.5).	Mortality rate 7.3 deaths/1,000 (5.2–10.2)	6.41 deaths/1,000 (4.4–9.4)

a *range*.

b *mean or median*.

#### Risk of Mortality by Age and Sex

Four studies reported a significantly higher risk of mortality in younger children (33, 36, 38, 39), and mortality was highest among teenagers only in one study (40). Age was not associated with mortality in two reports (37, 41), and the age-mortality association was not reported in two studies (Table 2). The risk of mortality comparing boys and girls with epilepsy was similar in most studies (Table 2). Only one study (40) reported a significantly higher risk among boys (HR 1.3, [95% CI 1.1–1.5]).

#### Risk of Mortality by Epilepsy Factors

Most deaths (68.8%) occurred in the first 10 years after epilepsy diagnosis, and mortality declined significantly in the subsequent decades but remained higher compared with the general populations (36, 38, 39). Mortality was higher in structural/metabolic epilepsy compared with epilepsy of genetic or unknown etiology in three studies (34, 35, 41). The risk of mortality was also very high in epilepsy with comorbid brain disorders, some of which were possible causes of epilepsy (Supplementary Table 2).

Generalized seizures were associated with an increased risk of mortality at the univariable level, but not at the multivariate level (41). Mortality was increased in epilepsy syndromes such as Lennox-Gastaut syndrome and infantile spasms in one report (36). Most reports included in this review, however, did not report the effect of seizure type on mortality (Supplementary Table 2).

Mortality was also higher in: (i) epilepsy patients using more than 2 antiepileptic drugs (AEDs) (33, 39); (ii) patients without a 5 year terminal remission after treatment with AEDs (41); (iii) patients from rural settings (40); and (iv) patients with Medicare health insurance plan compared with private/commercial insurance in one US-based study (40).

#### Causes of Death in Epilepsy

Most deaths (median percentage 87.5% [range 74.9–90.6]) were caused by non-epilepsy-related causes such as underlying neurological disorders or respiratory problems (Table 3); but infections, tumors, cardiovascular disorders, and injuries were also reported in other studies (36, 40). Status epilepticus and sudden unexplained death in epilepsy (SUDEP) were the most common causes of epilepsy-related mortality (Table 3).

**Table 3:** Proportionate mortality or cause-specific mortality rates/ratios for epilepsy and non-epilepsy related causes.

**Study**	**Epilepsy-related**	**Epilepsy-unrelated**	**Epilepsy-unrelated sub-categories**	**Unknown**
Ackers et al. (33)	18 (11.9%)	110 (72.8%)	Underlying neurological disorders (110)	23 (15.2%)
Berg et al. (34)	2 (15.4%)	10 (76.9%)		1 (7.7%)
Callenbach et al. (35)	None	9 (88.9%)	Respiratory problems (8) transtentoria and brain herniation (1)	1 (11.1%)
Camfield et al. (37)	2 (7.7%)	24 (92.3%)	Pneumonia (14); infection or sepsis (3), suicide (2); shunt malfunction (1); pulmonary embolism (1); congestive heart failure (1); gastroesophageal reflux and failure to thrive (1); and homicide (1)	None
Christensen et al. (38)	None	766 (95.4%)		37 (4.6%)
Nickels et al. (39)	2 (12.5%)	14 (87.5%)		None
Sillanpaa and Shinnar (41)	33 (55%)	26 (43%)	Pneumonia (12)	1 (2.0%)
Autry et al. (36)	Number/proportion not reported	Not reported	Cause specific SMR for neurological causes 19.4 (10.0–33.8); Infections and tumors 7.4 (5.4–9.9); Cardiac deaths 3.4 (0.7–9.9)	Not reported
Selassie et al. (40)	2.8/1,000 person-years of observation (pyo)	Not reported	Developmental conditions: 5.9/1,000 pyo; cardiovascular disorders excluding congenital malformations 4.4/1,000 pyo; injuries from external causes 3.8/1,000 pyo	Not reported

### Intellectual Disability

#### Overall Risk of Mortality

The median SMR was 2.9 (range 2.0–11.6) for ID; however, one study reported a HR of 6.1 (95% CI 5.3–7.0) after 25 years of follow-up, and two studies reported crude mortality ratios of 1.8 and 1.7, respectively (Table 4). There was no difference in the overall risk of mortality between studies classifying ID using the International Classification of Disease Ninth Revision (ICD-9) or earlier versions and the Diagnostic and Statistical Manual of Mental Disorders Fourth Revision (DSM-IV) or earlier versions compared with studies classified using the ICD-10 or DSM-5. Studies of ID mainly utilized data from population-wide or state-wide disability service providers linked retrospectively with registries of mortality. Information about the definition of ID and sources of mortality data for each report are provided in Supplementary Table 3.

**Table 4:** General study characteristics, overall risk, and sociodemographic risk factors for mortality in people with intellectual disability.

**Author and Year of publication**	**Country or state**	**Percent quality score (Class)**	**Cohort size**	Follow-up^*^ (years)	**Overall measure of mortality (95% CI)**	**Risk of mortality by age**	**Risk of mortality by sex**	
							**Females**	**Males**
Arvio et al. (42)	Finland	64 (2)	–	15	SMR 2.9 (2.9–3.0)	SMR 11.6 (95% CI 9.6–13.8) for <15 years; SMRs decreased with increasing age; SMR 2.0 (1.95–2.14) for >60 years	SMR 4.1 (4.0–4.3)	2.4 (2.3–2.4)
Bourke et al. (43)	Western Australia	91 (1)	10,593	25	Adjusted HR 6.1 (5.3–7.0)	aHR 6.0 (95% CI 4.8–7.6) for ages 1–5 years, 12.6 (9.0–17.7) for 6–10 years, and 4.9 (3.9–6.1) for 11–25 years.		aHR 0.8 (0.6–1.0) (male vs. female)
Florio and Trollor (44)	New South Wales, Australia	55 (2)	40,705	6	SMR 2.5 (2.3–2.6)	0–19 years mortality rate ratio (MRR)= 4.6, 20–49 years = 4.7, 50–69 years=2.6, 70–79 years=1.7, and 80+ years MR=0.8	SMR 4.3 (3.8–4.7)	2.5 (2.3–2.8)
Forsgren et al. (45)	Vasterbotten, Sweden	99 (1)	1,478	7	SMR 2.0 (1.7–2.3)	SMR 16 (10-24) for those 0–19 years	SMR 2.6 (2.0–3.3)	1.6 (1.2–2.0)
Lauer and McCallion (46)	New York, United States (USA)	73 (2)	–	3	MR 1.8	MRR was 5.9 for 18–24 years and 1.8 for those 75+ years.	Mortality rate 11.2/1,000	10.9/1,000
McCarron et al. (47)	Ireland	82 (1)	31,943	10	SMR 3.9 (3.7–4.0)	SMR 6.7 (5.9–7.5) for 0–19 years; SMR decreased with increasing age; SMR 2.7 (2.4–3.0) for 80+ years	SMR 4.9 (4.6–5.2)	3.1 (2.9–3.3)
Tyrer et al. (48)	Leister shire and Rutland, United Kingdom	91 (1)	2436	10	SMR 3.2 (2.9–3.6)	SMR 11.5 (8.1–15.8) in the 20s; diminished in older ages; SMR 1.5 (1.2–1.8) for 70+ years	SMR 3.6 (3.1–4.2)	2.9 (2.5–3.3)
Shavelle et al. (49)	California, USA	91 (1)	64,207	30	MR 1.7	5–19 years (MRR = 1.4); 20–39 years = 2.0; 40–59 years = 1.8; 60+ years = 1.3	Mortality ratio = 1 (females and males)	
Smith et al. (50)	Scotland	73 (2)	18,278	5	SMR 11.6 (9.6–14.0)	5–14 years SMR = 21.6 (16.6–28.2); ≥ 15 years but <25 years SMR = 7.7(5.9–10.2)	SMR of 16.6 (12.2–22.6)	9.8 (7.7–12.5)
Cooper et al. (51)	Glasgow, Scotland	91 (1)	961	17	SMR 2.2 (2.0–2.5)	SMR of 18.7 (0.4–37.1) for 15–25 years; 2 (1.3–7.1) for 26–35 years; 3.9 (2.3–5.4) for 36–45 years; 3.8 (2.9–4.7) for 46–55 years; and 1.9 (1.6–2.1) for >55 years	SMR 3.5 (2.9–4.1)	1.7 (1.4–2.0)

* *range*.

#### Mortality by Age and Sex

Mortality was highest in younger age-groups in all studies (Table 4). The median SMR for the ages 0–19 years was 13.8 (range 6.7–21.6). Most studies reported a monotonic decline of mortality ratios with increasing age, and mortality was slightly higher than the respective general populations in older age groups (60+ years), suggesting a healthy survivor effect (Table 4). The median SMR was 4.1 (range 2.6–16.6) for females and 2.5 (range 1.6–9.8) for males. Mortality was similarly high in males and females in a study excluding ID cases with significant physical impairment or comorbid/underlying degenerative conditions (49).

#### Mortality by Factors Related to Intellectual Disability

The risk of premature mortality was consistently higher in severe or profound ID compared with mild or moderate ID (Table 5). Three studies, however, did not report mortality by the severity of ID (44, 46, 50). Mortality in ID was significantly increased by genetic disorders (Down syndrome and Fragile X), maternal alcohol use, low-birth weight and postnatal injury, and the presence and number of comorbid neurological conditions such as epilepsy and cerebral palsy (43, 45, 48, 51) (Table 5).

**Table 5:** Mortality by severity and aetiology of intellectual disability.

**Author and Year of publication**	**Overall estimate of mortality (95% CI)**	**Risk of mortality by level of disability (95% CI)**						**Mortality by cause of ID or comorbid neurological disorders (95% CI)**
		**Mild**	**Mild or moderate**	**Moderate**	**Severe**	**Severe or profound**	**Profound**	
Arvio et al. (42)	SMR 2.9 (2.9–3.0)	2.3 (2.2–2.4)	–	–	3.4 (3.3–3.5)	–	–	–
Bourke et al. (43)	Adjusted HR 6.1 (5.3–7.0)	–	3.2 (2.6–3.9)	–	40.6 (33.4–49.2)	–	–	Biomedical causes 24.4 (20.7–28.7); unknown causes 1.8 (1.3–2.3); Autism 2.0 (0.8–4.7)
Florio and Trollor (44)	SMR 2.5 (2.3–2.6)	–	–	–	–	–	–	–
Forsgren et al. (45)	SMR 2.0 (1.7–2.3)	1.8 (1.1–2.7)		1.5 (1.1–2.0)	2.0 (1.5–2.6)		8.1 (5.6–11.7)	Mental Retardation 1.7 (1.4–2.0); MR + Epilepsy 5.0 (3.3–7.5); MR + Epilepsy + CP 5.8 (3.4–9.8)
Lauer and McCallion (46)	MR 1.8	–	–	–	–	–	–	–
McCarron et al. (47)	SMR 3.9 (3.7–4.0)	5.0%	–	7.8%	14.6%		24.8%	–
Tyrer et al. (48)	^*^SMR 3.2 (2.9–3.6)	–	–	–	–	–	–	Comorbidity with Downs Syndrome 7.60
Shavelle et al. (49)	Mortality ratio was 167%		165%	–	–	185%	–	–
Cooper et al. (51)	SMR 2.2 (2.0–2.5)	1.6 (1.3–1)	–	2.1 (1.6–2.6)	2.8 (2.1–3.4)	–	4.1 (3.1–5.2)	ID with Downs Syndrome 5.3 (4.0–6.6); ID without Downs Syndrome 1.9 (1.7–2.2)
Smith et al. (50)	SMR 11.6 (9.6–14.0)	–	–	–	–	–	–	–

* *The study by Tyrer et al. (48) excluded participants with mild ID; those with moderate to profound ID included as one group in the analysis*.

#### Causes of Death in Intellectual Disability

Respiratory infections (34%), accidents (18%), and epilepsy (10.7%) were the most common causes of death in ID in one study from Western Australia (43); while, the leading causes of death in a Swedish study were congenital malformations (SMR 46.3 [32.9–65.0]), neurological diseases (SMR 9.7 [5.5–17.0]), mental disorders (SMR 4.0 [1.9–8.4]), and respiratory diseases (SMR 3.3 [2.0–5.5]) (45). Diseases of the respiratory system (21.8%), the circulatory system (19.1%) and the nervous system (13.0%) were the most common causes of death in the Scottish study (51). Another Scottish study (50) identified diseases of the nervous system (33%), congenital malformations, deformations and chromosomal anomalies (22%), and nutritional, metabolic and endocrinal diseases (8%) as the most frequent causes of death. Six out of 10 (60%) studies did not report the causes of death in ID.

### Cerebral Palsy

#### Overall Risk of Mortality

We identified 3 studies of CP (52–54) which assessed the effect of motor impairment on the risk of mortality in children. The risk of mortality in CP was highest between the ages of 2–15 years compared to the general population rates (SMRs>25) and declined steadily to 2–3 times higher than the population rates by the age of 40 years (52, 54). The study from Bangladesh did not estimate the SMR but crude mortality rates (19.5 per 1,000 person-years) and mortality was highest in the youngest age group (<5 years).

#### Motor Impairment and Mortality in Cerebral Palsy

Lack of independent ambulation was the strongest predictor of mortality (adjusted HR 6.1, [3.3–11.8]) in the Australian study (54). However, motor impairment was a weaker predictor of premature mortality (MRR 1.4 [1.1–1.7]) than ID (MRR 2.1, [1.9–2.4]) in the Western Australian study (52). The latter study further reported that the overall disability score was a better predictor of mortality than motor impairment and ID, separately. While mortality was elevated among those with hearing impairment (adjusted HR 2.9 [1.2–6.7]) and swallowing difficulties (adjusted HR 2.3 [1.0–4.9]) in the study from Bangladesh (53), the risk of severe motor impairment (GMFCS levels III-V) on mortality did not reach statistical significance (adjusted HR 2.4 [0.7–8.4]).

#### Causes of Death in Cerebral Palsy

CP as an underlying cause of death was accountable for 79% of deaths in the Western Australian study with 59% of the fatalities directly resulting from respiratory problems (52). Similarly, respiratory causes were the most common direct causes of death in the Australian study (54). Meningitis (31.0%) and pneumonia (27.6%) were the leading causes of death in the study from Bangladesh (53) and most children who died were either severely malnourished or had feeding problems.

### Vision Impairment and Mortality

The study from Malmöhus, Sweden (55) reported an odds ratio for mortality of 60.11 (95% CI 35.2–97.9) in visually impaired children and adolescents compared with an age- and sex-matched sample from the population. Most of the visually impaired children had additional impairments such as CP and ID, and respiratory diseases were the most common cause of death in this study.

### Mortality in Multiple Domains of Childhood Neurological Impairment

The study from Kenya (14) investigated the long-term risk of premature mortality in children aged 6–9 years with NI in 5 domains (epilepsy and impairments in cognitive, hearing, vision and motor functions) compared with an age-matched sample from the general population. The overall risk of mortality was >3 times higher among those with any impairment compared to the general population (SMR 3.2 [1.7–5.5]). Developmental delay (adjusted HR 18.9, [2.2–160.4]) and severe malnutrition (20.9, [3.14–139.11]) increased the risk of mortality, and infections such as HIV and accidents were the most common causes of death.

## Discussion

The studies in this systematic review reported that the measures of mortality were significantly greater in children with NI compared with the general population. The estimates were greatest for CP (SMR > 25) and lowest for ID (SMR = 2.9), with few studies reporting mortality outcomes for visual impairment, and no data for hearing impairment. Clinical cohort studies had higher estimates than population-based cohort studies, probability of severity bias in the former. These estimates should be interpreted carefully since the methodology and follow-up periods differed across the studies, complicating the combining of estimates across studies. The risk of mortality following NI depends on younger age, the severity of the primary impairment and the number and severity of comorbid disorders. Most causes of the mortality including infections, cardiovascular diseases, and tumors were unrelated to NI.

### Interpretation for Epilepsy

Mortality was significantly higher in children with epilepsy compared with the reference population similar to a previous review of mortality in pediatric epilepsy (56), and so were the factors underlying mortality in NI (16, 19, 57). Early-onset epilepsy occurs in a period of vulnerability to adversities such as infections and severe epileptic encephalopathy, which can increase the risk of mortality (58). Structural epilepsy complicated by head injuries and infections of the brain has poor outcomes e.g., mortality, particularly in LMICs (16). Mortality risks are highest in intractable epilepsy with reduced responsiveness to AEDs (19). Mortality was generally similar in both males and females, although few studies reported high rates in males, probably related to their vulnerability to accidents from injuries and violence (40). Mortality risk reduces in subsequent decades (59); implying that those surviving may have responded better to AEDs or had the fewest risk factors for mortality. There are other context-specific factors for increased mortality such as residence and type of insurance which are related to social, economic and cultural disadvantages that influence access to AED treatment.

Lastly, our findings concur with previous studies that most deaths are caused by underlying comorbid brain disorders and respiratory infections and not by seizures or epilepsy (57, 60). Comorbidity conditions occur more frequently in those with severe epilepsy, who are more likely to die (61). Reducing mortality requires the management of both epilepsy and other comorbid conditions. Risk is elevated in generalized tonic seizures, which are often reported by many who die from SUDEP (62, 63), a condition that is not accurately documented in LMICs because coroner's autopsy reports are unavailable. Like SUDEP, status epilepticus was an infrequently identified cause of mortality compared to non-epilepsy causes, yet it occurs in 25–30% of people with epilepsy (64) and is known to increase mortality in children (16, 65). In many LMICs, status epilepticus is a complication of endemic infections that should be additionally prevented or managed (66) and may be due to poor access to AEDs, inappropriate treatment and delayed initiation of treatment (67). Most reported causes of death were non-epilepsy related e.g., cardiovascular problems, and respiratory problems, and so a comprehensive care and public health plan for children with epilepsy that includes other medical conditions is advised, especially in settings where these are endemic.

### Interpretation for Intellectual Disability

This review corroborates findings from a previous review (18) that the overall risk of mortality in people with childhood-onset ID is higher than the general population. Mortality risk was, however, lower than that for epilepsy, which might imply better care for ID patients in HICs, use of cohorts of milder ID, or misreported due to stigma. Underreporting of deaths due to stigma related to ID intellectual disability not only underestimates the prevalence in general but also reduces the SMRs or relative risk for deaths related to ID if these deaths are classified under the general population. There was no single study of ID from LMICs, where prevalence and associated mortality may be higher (68). A median SMR of 2.9 for ID is probably an underestimate because a majority of the studies utilized a retrospective design which may be subject to three types of bias: (i) over-representation of severe ID in clinic samples; (ii) loss to follow-up bias and; (iii) bias due to incomplete data linkage for follow up which might under-uncertain the risk of mortality artificially lowering the SMRs (69). The risk of mortality is highest in the youngest age-groups, decreasing steadily with age, which is well-appreciated in the literature (70–72). The SMRs are higher in females than males, probably consistent with inequality in access to care (18). The risk of mortality is higher in severe-profound ID, which expectedly would be associated with significant functional limitation and disability.

Genetic causes of ID were important risk factors for mortality, and Fragile X syndrome was not exceptional in this review, with a remarkable reduction in life expectancy compared with general populations. It is known that neuropsychiatric problems like epilepsy are very common in ID (73–75) and can increase mortality. Pregnancy and perinatal factors were important risk factors suggesting they will not only cause ID but will worsen its prognosis including premature mortality. Noteworthy was maternal drug abuse and mortality in ID which can be explained by poor parenting of the affected children (76). Children with ID are susceptible to accidents that can be fatal and safety measures and close supervision are encouraged. Respiratory infections are important causes of morbidity and mortality which should be prevented and managed to improve outcomes in children with ID.

### Interpretation for Motor Impairment in Cerebral Palsy

Cerebral palsy (CP) had the greatest risk of mortality, which is well-recognized in other studies (77–79). Motor impairments and ID were the strongest predictors of premature mortality in CP in the studies included in this review; both comorbidities are debilitating complications of CP. Two previous studies (80, 81), concur that additional neurological comorbidity worsens the disability score significantly reducing the chances of survival among children with CP. Disability scores, therefore, offer a better prediction of survival compared with motor impairment and/or ID, separately. Rehabilitative therapies to manage physical impairment while optimizing the mobility of children with CP may improve the quality of life and reduce the risk of mortality (82, 83). Respiratory infections are the most common direct causes of death perhaps because CP impairs breathing and respiratory hygiene. Studies from LMICs settings highlight the significance of preventing other infections such as HIV, pneumonia, and meningitis as well as severe malnutrition to improve CP outcomes.

### Interpretation for Vision and Hearing Impairment

Mortality is higher among visually impaired children having comorbid and severe neurological disorders, but the study did not provide SMRs, affecting comparisons with the general population. Only cause-specific estimates of mortality would separate the effects of loss of sight on mortality from the comorbid disorders, but these data were not available for this review. The risk of mortality for visual impairment alone was not significantly higher in the Kenyan study, where children with five domains of NI were followed for over 14 years to determine mortality, which was due to small numbers. There was no single study on hearing impairments and associated all-cause mortality, which could be due to publication bias (4) or because this impairment is often overlooked to follow up of mortality outcomes. In this review, children with CP had hearing impairments which increased the risk of premature mortality (53), suggesting the need to give attention to morbidity and disability of deaf children.

### Limitations

The distribution of the studies identified by our searches was sparse, with most from HICs such as Europe and North America with fewest in Asia and Africa and none from South America. This may affect the generalizability of these estimates across continents or other countries, where specific studies are needed. The follow-up periods were variable, whereby studies with shorter follow-ups may underestimate the true burden of premature mortality. Follow-up times were reported differently in the primary studies, for instance, most studies neither reported the mean nor the median follow-up duration in years. This differential reporting of the follow-up duration hindered the estimation of weighted median SMRs. There is often incomplete documentation and certification of deaths due to a lack of functional vital registration systems, which affects case ascertainments yielding lower estimates of mortality. Because the grading process involved qualitative judgment by the reviewers, the interpretation of study methods and the application of criteria might be inconsistent and unreliable. The study populations from which we obtained the data vary greatly in terms of population characteristics, health, and social systems. Clinical-based estimates may overrepresent severe forms of NI that have additional risk for mortality; most LMICs have limited resources to do follow-up studies of mortality, often doing these studies in high-risk zones that are not representative of other low-risk areas. Despite these limitations, our review obtained critical evidence that might increase the survival of children with childhood-onset NI and disability.

## Summary

The risk of premature mortality is elevated in children with NI, or adults with childhood-onset NI, it being higher in CP and epilepsy, and lower in ID. There are few SMR studies for visual, hearing, and motor impairments. We recommend future population-based follow-up studies for multiple domains of NI in children, especially for those with visual, hearing, and motor impairments, and in LMICs where there is a dearth of evidence. Few studies in this review originated from LMICs, yet these countries have a concentration of risk factors and the highest burden of childhood NI and disability. The similarity of risk factors and causes of death across the five domains of NI provides an opportunity for integration of preventive, curative and rehabilitative services. Integrated interventions targeting modifiable risk factors, for instance, improving access to AEDs and prompt treatment of childhood epilepsy as well as caregiver/parental training, child supervision, and prevention of respiratory infections for children with ID, immunization, and improved nutrition in LMICs, are required to improve survival and quality of life among the affected children and families. It worth advising the families that premature death among children cannot be preventable in the presence of genetic causes for NI conditions that are untreatable and progressive such as mucopolysaccharidosis.

## Data Availability Statement

The original contributions presented in the study are included in the article/Supplementary Material, further inquiries can be directed to the corresponding author/s.

## Author Contributions

JA: protocol development, searches, identification, appraisal of eligible studies, data synthesis, and writing the first draft of the manuscript. SKa: protocol development, searches, identification, and subsequent review of drafts. SKi: protocol development and review of subsequent drafts. MB: protocol development and review of subsequent drafts. CN: protocol development and review of subsequent drafts. All authors: have read and approved the final manuscript.

## Conflict of Interest

The authors declare that the research was conducted in the absence of any commercial or financial relationships that could be construed as a potential conflict of interest.
