# Supplementary material for: Premature Mortality, Risk Factors, and Causes of Death Following Childhood-Onset Neurological Impairments: A Systematic Review
Source: Front Neurol. 2021 Apr 9;12:627824. doi: 10.3389/fneur.2021.627824 (PMC8062883; doi:10.3389/fneur.2021.627824)
Supplement: Supplementary file 1 [file Table_1.docx]

**Supplementary Table 1: Study characteristics and overall mortality estimates of the identified cohort studies in the systematic review**

| **Author & Year of publication** | **Country** | **Study setting** | **Cohort design** | **Domain** | **Quality**  **(class)** | **Cases** | **Number of deaths** | **Measure of mortality (95% CI)** |
| --- | --- | --- | --- | --- | --- | --- | --- | --- |
| (Ackers et al., 2011) | UK | Clinical | Retrospective | Epilepsy | 2 | 6,190 | 151 | SMR 22.4 (18.9-26.2) |
| (Arvio et al., 2016) | Finland | Population | Retrospective | ID | 2 | - | 5,171 | SMR 4.3 (4.2-4.4) |
| (Autry et al., 2010) | US (Atlanta) | Clinical | Retrospective | Epilepsy | 1 | 688 | 64 | SMR 3.1 (2.4-4.0) |
| (Berg et al., 2004) | US (Connecticut) | Clinical | Prospective | Epilepsy | 1 | 613 | 13 | SMR 7.5 (4.4-13.0) |
| (Blohme and Tornqvist, 2000) | Sweden | Clinical | Retrospective | VI | 2 | 128 | 17 | OR 60.1 (35.2-97.9) |
| (Bourke et al., 2017) | Australia | Population | Retrospective | ID | 1 | 10,593 | 326 | HR 6.1 (5.3-7.0) |
| (Callenbach et al., 2001) | Netherlands | Clinical | Prospective | Epilepsy | 1 | 472 | 9 | SMR 7.0 (2.4-11.5) |
| (Camfield et al., 2002) | Canada | Clinical | Retrospective | Epilepsy | 1 | 686 | 26 | SMR 7.1 (3.2-10.9) |
| (Christensen et al., 2015) | Denmark | Population | Retrospective | Epilepsy | 1 | 25,244 | 803 | MMR 14.9 (13.9-16.1) |
| (Florio and Trollor, 2015) | Australia | Population | Retrospective | ID | 2 | 40,705 | - | SMR 3.2(2.9-3.4) |
| (Forsgren et al., 1996) | Sweden | Population | Retrospective | ID | 1 | 1,478 | 124 | SMR 2.0 (1.7-2.3) |
| (Lauer and McCallion, 2015) | US | Population | Retrospective | ID | 2 | - | - | MR 1.8 |
| (McCarron et al., 2015) | Ireland | Population | Retrospective | ID | 1 | 31,943 | 2,666 | SMR 3.9 (3.7-4.0) |
| (Nickels et al., 2012) | US | Clinical | Retrospective | Epilepsy | 2 | 467 | 16 | SMR 9.0 (5.4-14.4) |
| (Reid et al., 2012) | Australia | Population | Retrospective | MI in CP | 2 | 3507 | 418 | - |
| (Selassie et al., 2015) | US | Clinical | Retrospective | Epilepsy | 1 | 13,098 | 447 | HR 3.8 (3.1-4.7) |
| (Sillanpaa and Shinnar, 2010) | Finland | Clinical | Prospective | Epilepsy | 1 | 245 | 60 | SMR 6.4 (5.9-7.0) |
| (Tyrer et al., 2007) | UK | Clinical | Retrospective | ID | 1 | 2436 | 409 | SMR 3.2 (2.9-3.6) |
| (Shavelle et al., 2014) | USA | Population | Retrospective | ID | 1 | 64,207 | 1,514 | MR 1.7 |
| (Blair et al., 2001) | Australia | Population | Retrospective | MI in CP | 1 | 2014 | 225 | - |
| (Jahan et al., 2019) | Bangladesh | Population | Prospective | MI in CP | 2 | 678 | 29 | - |
| (Abuga et al., 2019) | Kenya | Population | Prospective | Epilepsy, ID, VI, HI, MI | 1 | 284 | 11 | SMR 3.2 (1.7–5.5) |
| (Cooper et al., 2020) | Scotland | Population | Retrospective | ID | 1 | 961 | 294 | SMR 2.2 (2.0-2.5) |
| (Smith et al., 2020) | Scotland | Population | Retrospective | ID | 2 | 18,278 | 106 | SMR 11.6 (9.6-14.0) |

ID=intellectual disability; VI=Vision impairment; MI=Motor impairment; MI in CP=motor impairment in cerebral palsy; HI=Hearing impairment.
