# Supplementary material for: Premature Mortality, Risk Factors, and Causes of Death Following Childhood-Onset Neurological Impairments: A Systematic Review
Source: Front Neurol. 2021 Apr 9;12:627824. doi: 10.3389/fneur.2021.627824 (PMC8062883; doi:10.3389/fneur.2021.627824)
Supplement: Supplementary file 2 [file Table_2.docx]

**Supplementary table 2: Risk of mortality by epilepsy-specific factors**

| **Study** | **Mortality in structural/metabolic epilepsy** | **Mortality in genetic or epilepsy of unknown etiology** | **Mortality in Comorbid neurological conditions** | **Mortality following generalized seizures** | **Mortality after focal seizures** | **Mortality after status epilepticus** | **Mortality in epileptic syndromes** |
| --- | --- | --- | --- | --- | --- | --- | --- |
| Ackers (2011) (Ackers et al., 2011) |  |  | 123 of 151 (81.5%) children who died had an underlying neurological or metabolic disorder |  |  |  |  |
| Berg (2004) (Berg et al., 2004) | SMR of 33.5 (18.5-60.4) in symptomatic epilepsy; adjusted HR of 10.2 (2.1-49.6) for remote symptomatic epilepsy & 13.3 (3.4-51.7) for epileptic encephalopathy | The SMR was 1.4 (0.4-5.7) for non-symptomatic epilepsy |  |  |  | Not associated with death in the final multivariable model |  |
| Callenbach (2001) (Callenbach et al., 2001) | All 9 deaths occurred in symptomatic epilepsy (SMR 22.9, [5.8-37.9]). | No deaths recorded among children with non-symptomatic epilepsy (SMR 0, 0-2.2) |  |  |  |  |  |
| Autry (2010) (Autry et al., 2010) |  |  | MR of 6.4 (4.6-8.5). for co-occurring disabilities: MR 2.9 (1.5- 5.3) for 2; MR 7.5 (4.5-11.7) for 3; MR 18.5 (10.1- 31.1) for 4; Isolated epilepsy MR 1.41 (0.85-2.19) |  |  |  | Lennox-Gastaut syndrome (MR 13.9, [7.2-24.3]);  Infantile spasms (MR 11.9, [5.1-23.5]) |
| Camfield (2002) (Camfield et al., 2002) |  |  | Adjusted RR 22.0 (7.0-69.7) for severe comorbid neurological disorder | Secondary generalized (SG) Multivariate RR of 0.65 (95%CI 0.25-1.68) | Partial seizures Multivariate RR of 0.75 (0.75-8.05) f |  |  |
| Christensen (2015) (Christensen et al., 2015) |  |  | MMR was 10.9 (9.7-12.1) after excluding adverse birth outcomes; and 4.2 (3.3-5.3) after excluding adverse outcomes + neurological disorders |  |  |  |  |
| Nickels (2012) (Nickels et al., 2012) |  |  | multivariable HR 12.8 (1.4-117.0) for abnormal neurological exam | Univariable RR 0.7 (0.2-2.5) | Univariable RR 1.37 (0.44-4.24) | Univariable HR 4.4 (1.6-11.9);  Multivariable HR 1.34 (0.48-3.77) |  |
| Selassie et al., 2015 (Selassie et al., 2015) |  |  |  |  |  |  |  |
| Sillanpaa (2010) (Sillanpaa and Shinnar, 2010) | A remote symptomatic cause was associated with increased risk of death compared with cryptogenic/idiopathic cause (37% vs.12%, P<0.001) | Cryptogenic/idiopathic epilepsy was the reference |  |  |  | Univariable HR 2.1 (1.1-7.0); multivariable HR 1.5(0.7-3.0) |  |
