# Supplementary material for: Premature Mortality, Risk Factors, and Causes of Death Following Childhood-Onset Neurological Impairments: A Systematic Review
Source: Front Neurol. 2021 Apr 9;12:627824. doi: 10.3389/fneur.2021.627824 (PMC8062883; doi:10.3389/fneur.2021.627824)
Supplement: Supplementary file 3 [file Table_3.docx]

**Supplementary Table 3: Identification of study participants, case definitions, and source of mortality data for studies on Intellectual Disability**

| **Author & Year of publication** | **Identification of people with Intellectual Disability (ID)** | **Definition/classification of Intellectual Disability** | **Level of Intellectual Disability** | **Determination of mortality and causes of death** |
| --- | --- | --- | --- | --- |
| (Arvio et al., 2016) | The national social insurance institution (KELA) provided a list of individuals who received disability benefits due to ID | ID defined based on the ICD-10 criteria (WHO 1996): an IQ< 70; presence of age-inappropriate adaptive skills; and a clinical manifestation during the developmental stages | Mild (IQ 50-69); severe (IQ<50) | Finnish population Register Centre delivered mortality data to KELA determined by the termination of benefits due to death |
| (Bourke et al., 2017) | Population-based register of all live births linked  to the Intellectual Disability Exploring Answers (IDEA)  database to identify children with ID | IQ<70 or deficits in adaptive behavior occurring before the age of 18 years or having a known condition consistent with ID e.g. Downs syndrome | Mild (IQ 55-69); moderate (IQ 40-54); severe (IQ <40) | Western Australian Mortality Database (ICD-10 classification) and Australian Bureau of Statistics; autopsy reports used to determine the cause of death among ID cases |
| (Florio and Trollor, 2015) | New South Wales Department of Ageing, Disability and Home Care (ADHC) service register for people with ID was used | DSM-IV criteria in the definition and classification of ID | - | Mortality data were obtained from the registry of births, deaths and marriages in the New South Wales |
| (Forsgren et al., 1996) | Prevalence study of all cases with mental retardation | ID defined as people with IQ<70; present before the age of 18 | Mild mental retardation (MR) IQ 50-55 to 70; moderate IQ 35-40 to 50-55; severe IQ 20-25 to 35-40; and profound IQ<20-25 (DSM-III) | The Swedish National Central Bureau of Statistics, underlying and contributory cause of death; death certificates were also evaluated. |
| (Lauer and McCallion, 2015) | US state intellectual and developmental disabilities service systems used to identify study participants | Definition of ID Varied by state and environments: The ICD-9 was used in medical environments & DSM-IV in mental health environments. ICD-9: the presence of neurodevelopmental disorders associated with ID and IQ<70; the DSM-IV used a combination of criteria involving IQ<70, age at onset <18 years and presence of functional impairments | ICD-9 code used to classify ID as mild, moderate, severe or profound. | An external data system e.g. US Social Security Death Index; internal systems such as billing systems; and State Department  of Health death certificates were used in the determination of death. |
| (McCarron et al., 2015) | Data from national intellectual disability database of Ireland (NIDD) provided ID cases | Not explained | Not explained | Central Statistics Office provided data  on deaths. |
| (Tyrer et al., 2007) | Leicestershire  Learning Disability Register was used to identify adults having moderate-severe Intellectual Disability (ID) excluding mild cases of ID | Enrolment to the register was based on a moderate, severe or profound developmental  intellectual impairment (WHO 1992) with adaptive behavior problems, and the likely need for long-term support. | Moderate IQ 35-49; severe IQ 20-34; and profound IQ <20. | People enrolled on the register and died were identified using mortality data  from the Office of National Statistics. |
| (Shavelle et al., 2014) | Database of the California department services for people with ID, US identified people using the services; ID cases with significant physical impairment, comorbidity, or underlying degenerative conditions were excluded | ID was defined as the presence of limitations in intellectual functioning and adaptive behavior before the age of 18 years | Mild IQ 50-70,  moderate 35-50, severe 20-35, profound <20 | Mortality data were obtained from the California Department  of Health Services, Bureau of Vital Statistics |
| (Cooper et al., 2020) | Participants identified through general practitioners’ registered patients, health and social work services, health board registers, payment records by social services | ID defined and classified according to the ICD-10 criteria | Classified as mild, moderate, severe and profound; specific IQ values for classification not provided | Mortality data obtained through data linkage of existing data with the National Records for Scotland death certification data. |
| (Smith et al., 2020) | Educational data from Scotland’s annual pupil census between 2008 and 2013 where children with additional support needs due to intellectual disability were identified. | Not defined | Not classified | Individual data linkage with the National Records of Scotland deaths register. |
